# Editorial: Translation of Genomic Results Into Public Health Practice

**DOI:** 10.3389/fpubh.2018.00156

**Published:** 2018-05-25

**Authors:** Natasa Djordjevic, Stefania Boccia, Róza Ádány

**Affiliations:** ^1^Department of Pharmacology and Toxicology, Faculty of Medical Sciences, University of Kragujevac, Kragujevac, Serbia; ^2^Section of Hygiene, Institute of Public Health, Università Cattolica del Sacro Cuore, Fondazione Policlinico ‘Agostino Gemelli’ IRCCS, Rome, Italy; ^3^Department of Preventive Medicine, Faculty of Public Health, University of Debrecen, Debrecen, Hungary

**Keywords:** genomics, public health practice, public health, implementation, genetics

First genetic theories of inter-individual variability in disease susceptibility and therapy response date back to more than a century (1). Since then, genomic knowledge, accumulated and consolidated through the years of studies, paved the way for the new era of healthcare, where the interest is shifted from an “average patient”-oriented general approach to the precision medicine, i.e., diagnostics and treatment tailored for an actual individual (2–4). Society, on the other hand, still remains the main concern of public health, long ago defined as “the art and science of promoting health and preventing disease” at the community level (5). Nowadays, when the two fields converge at the common aim of achieving a healthier population, the genomic information offers the opportunity of proactive rather than reactive approach in reaching the goals of modern medicine (6–8).

The timely diagnostics, the individualized treatment, and the disease prevention and control could be in the hands of the public health genomics (9–12), defined as the “effective and responsible translation of genome-based knowledge and technologies for the benefit of population health” (13). Yet, the implementation of genomic results seems to be relatively slow, leaving the gap between the scientific discovery and the clinical utility (2, 14). The Frontiers research topic entitled “Translation of genomic results into public health practice” was brought forward to provoke the scientific community to contribute to the assessment of the current state of the public health genomics. The submitted papers lived up to our expectations, addressing the most important aspects of the topic, warning of the challenges, and limitations we are facing, but also giving the evidence of effectiveness and recommending the optimal methods and future steps in the process of translation of genomic results into public health practice.

A public health perspective of the barriers and facilitating factors for implementation of genetic services was given by Cornel and van El. While acknowledging several promising examples of the genetic services, the authors keep the focus on the work yet to be done, providing the summary of the most important obstacles to overcome, and reminding of the functional aids for genomic results to be translated into public health practice. An insight into emerging legal and ethical issues related to application of genetic testing in health care systems, provided by Sándor, brings attention to the two very important aspects of genetic information. In the paper, author points out the challenges personalized medicine is currently facing in private and public sector, exploring the legal framework required for integration of genetic testing into routine practice. Unim et al. presented the protocol for a multicenter qualitative study in identification of delivery models for the provision of predictive genetic testing in Europe. The estimation of health community's readiness to incorporate public health genomics into their practice and the evaluation of the currently available delivery models are necessary steps in optimization and standardization of genetic service, with an aim to provide recommendations to decision makers in relation to transfer of genomic technologies from research to clinical application. Fiatal and Adany summarized the latest development in the field of cardiovascular genetics, spanning from gene discovery to an evidence-based health application with population health impact. In the systematic review of the studies from the last decade, the authors estimated the applicability of genetic data in identification of increased risk for common cardiovascular diseases, and identified the research gaps where additional investigations are warranted. Tognetto et al. delivered a systematic review on the existing screening pathways for identification of the most common hereditary colon cancer syndrome known as Lynch syndrome, revealing genetic testing as one of the most crucial diagnostic tools, and genetic counseling as an irreplaceable step in the process of colorectal cancer risk assessment and prophylaxis. In another systematic review, written by Migliara et al., the guidelines on genetic testing and patient management in familial hypercholesterolemia were assessed, disclosing the currently recommended criteria that identify subjects requiring genetic testing, and the health-care pathways available for those with positive results. Finally, Mišić et al. reported the prevalence of genotypes that determine the resistance of Staphylococci to macrolides and lincosamides in Serbia. The study stresses the importance of genetic testing and genetic monitoring for timely discovering of resistance to antibiotics, with an aim to avoid treatment failure in patients with serious staphylococcal infection.

The presented papers, assembled in the joint research topic, recognize the translation of genomic results into public health practice as a frontier of the contemporary medicine. Numerous challenges lie ahead, yet the ways of dealing with them are recognized and achievable. With all the available evidence of their clinical validity and utility, and a growing awareness of their importance for public health, we believe reliable and actionable genomic results are bound to become an inseparable part of routine medical practice worldwide.

## Author contributions

ND, SB, and RA jointly contributed to the topic development and the manuscript preparation.

### Conflict of interest statement

The authors declare that the research was conducted in the absence of any commercial or financial relationships that could be construed as a potential conflict of interest.
